# The relationship between immigration status and chronic kidney disease risk factors in immigrants and US-born adults

**DOI:** 10.1007/s10903-020-01054-x

**Published:** 2020-12

**Authors:** Aprill Z Dawson, Emma Garacci, Mukoso Ozieh, Rebekah J Walker, Leonard E Egede

**Affiliations:** 1 Department of Medicine, Division of General Internal Medicine, Medical College of Wisconsin, 9200 W. Wisconsin Ave., Milwaukee, WI 53226; 2 Center for Advancing Population Science, Medical College of Wisconsin, 8701 Watertown Plank Rd, Milwaukee, WI 53226; 3 Department of Medicine, Division of Nephrology, Medical College of Wisconsin, 9200 W. Wisconsin Ave., Milwaukee, WI 53226

**Keywords:** Immigrants, Chronic Kidney Disease, Estimated Glomerular Filtration Rate, Albumin-to-Creatinine Ratio

## Abstract

**Objective:**

To understand the relationship between nativity and measures of kidney function including estimated glomerular filtration rate (eGFR) and albumin-to-creatinine ratio (ACR).

**Methods:**

Seven waves of data from the National Health and Nutrition Examination Survey (2001 – 2014) was analyzed. General linear regression methods were used to assess the relationship between eGFR, ACR and nativity (foreign-born vs US-born). Models were adjusted for length of time in the US, demographic variables, comorbidities, lifestyle factors, and access to healthcare.

**Results:**

There were 27,111 individuals representing 217,842,257 US adults included in the study. Approximately 26.1% were immigrants, with 40.4% of immigrants having resided <15 years in the US. Among immigrants with <15 years of residence, 51% were Hispanic, and 54.4% had high school or below education. After controlling for demographics and length of time in the US, immigrants were 26% more likely to have an ACR >= 30mg/g (OR=1.26, 95% CI: 1.08 – 1.47); however, after controlling for demographics, length of time, comorbidities, and lifestyle factors the results were no longer significant. Immigrants were significantly less likely to have an eGFR < 60 (OR=0.42, 95%CI: 0.36 – 0.50), which remained after adjustment (OR=0.75, 95%CI: 0.61 – 0.93).

**Conclusions:**

Immigrants had significantly lower odds of having an eGFR < 60 compared to US-born adults. Additionally, immigrants with >= 15 years in the US had mean eGFR values that were less than immigrants < 15 years in the US, indicating that there is some decrease in kidney function as the length of US residence increases.

## Introduction

There are about 30 million adults in the United States (US) (15%) with chronic kidney disease (CKD) and research suggests the prevalence is projected to increase to 16.7% by 2030 [1 – 5]. Estimated glomerular filtration rate (eGFR) and albumin-to-creatinine ratio (ACR) are widely accepted markers of kidney function and damage respectively. Estimated glomerular filtration rate less than 60 ml/min/1.73m^2^ lasting three months or more, or ACR greater than 30 mg/g for more than three months is indicative of CKD [6 – 8]. Additionally, eGFR greater than 60 with an ACR greater than 30 lasting three months or more is also indicative of kidney disease [8]. Individuals with kidney disease are at risk of developing complications like high blood pressure, anemia, and nerve damage [9]. The financial burden of CKD may reach up to $12,700 per person annually depending upon disease progression, with annual CKD costs for Medicare recipients totaling approximately $49 billion [10]. Early detection and treatment of CKD can prevent progression of disease, preventing the burden of kidney failure, dialysis, and the need for kidney transplant or end-stage renal disease (ESRD) [4, 9].

Vulnerable populations including seniors, minorities, and immigrants are at an increased risk of developing chronic diseases, however little is known about immigrants’ risk of CKD [9, 11]. More than 43 million immigrants were living in the US in 2015 [12]. While immigrants often arrive to the US in better health than that of the native-born population, research has shown that immigrant health declines the longer an individual resides in the US [13 – 16]. Immigrants tend to have lower levels of education, are less likely to have insurance, and are more likely to live in poverty compared to US-born individuals [12, 17].

Due to being uninsured and limited English proficiency, immigrants are frequently forced to delay and underutilize care needed to manage their chronic disease [18, 19]. It is known that immigrants with ESRD who do not have access to standard hemodialysis have 14 times increased risk of mortality compared to those with access [20]. Studies have also shown 15% of Hispanics are estimated to have CKD, and are 35% more likely than non-Hispanics to develop ESRD [4, 21]. Other studies have shown that NHB have significantly higher risk of developing ESRD compared to non-Hispanic White (NHW) patients [22, 23]. While information on differences in CKD among minorities and NHW exists, there is very little information about immigrants and CKD, and how they compare to NHW or other native-born populations [21]. Due to the gap in knowledge around CKD and immigrants, there is a need for more research to identify and address possible disparities in this area. Therefore, the aim of this study was to understand the relationship between immigration status and measures of kidney function including estimated glomerular filtration rate (eGFR) and albumin-to-creatinine ratio (ACR).

## Methods

### Data Source

The National Health and Nutrition Examination Survey (NHANES) is a series of cross-sectional surveys that were designed to estimate the health and nutritional status of adults and children in the United States [24]. NHANES includes interview survey questions on health and nutrition and a physical exam with laboratory testing, and releases data in two-year intervals [24]. NHANES over-samples certain groups such as ethnic minorities, low income, and elderly persons [24]. This study used data from continuous NHANES 2005–2014, among individuals 20 years of age and older, who completed both survey interview and physical examination. In total, 27,446 participants were selected. There were 16 participants without birth country information and were excluded, leaving the sample with 27,430 individuals valid for analysis.

### Variables

#### Country of Birth and Length of Time in US

Participants were asked “In what country were you born?”, the answers were grouped into born in the US or other country. If the individual answered born outside the US, then participants were asked to indicate the length of time they have resided in the US. Length of time in the US was grouped into < 15 years or >= to 15 years for those who were foreign-born. These cut points were selected based on the literature showing immigrant health declines as length of residence increases, with those who have lived in the US 15 years or longer having six times the odds of experiencing change in health compared to those who have resided in the country less than a year [13 – 15]. Research shows that immigrants living in the US 15 years or longer experience negative changes in health and tend to have health outcomes that are similar to the US-born population of a similar socioeconomic status [14]. Due to this previous research, length of residence was dichotomized into <15 years or >=15 years.

#### Albumin-to-Creatinine ratio (ACR) and Estimated Glomerular Filtration rate (eGFR)

Urinary albumin and urinary creatinine are measured in a random urine collected in the mobile examination center (MEC). The random urine albumin (URXUMA) in ug/mL and urine creatinine (URXUCR) in mg/dL were converted to the albumin/creatinine ratio (URDACT) in mg/g: URDACT = URXUMA/URXUCR × 100, round to .01. ACR was categorized as <30 (normal to mild increase) or ≥30 (moderate to severe) based on Kidney Disease Improving Global Outcomes (KDIGO) guidelines [8]. Estimated glomerular filtration rate was calculated using the CKD-EPI equation and the recommended formulae for eGFR per the KDIGO guidelines [8]. eGFR was categorized as >60 (normal to mild decrease) or ≤60 (moderate to severe decrease) based on KDIGO guidelines [8]. Creatinine from the serum specimen collected in the MEC was used to calculate eGFR [25].

#### Comorbidities

Comorbidities included hypertension, major adverse cardiac event (MACE), and depression. Hypertension was defined by the question: “Have you ever been told by a doctor that you had high blood pressure?” MACE was defined by any of the following been told by a doctor that the participant had: stroke, heart attack, angina, coronary heart disease, congestive heart failure. Depression was measured using the Patient Health Questionnaire (PHQ-9), a nine-item screening instrument that asks questions about the frequency of symptoms of depression over the past 2 weeks. Responses were given a point ranging from 0 to 3. Depression was coded as no depression (score 0–9), depression (score 10–27), and unknown (with missing score) [26].

#### Lifestyle Factors and Body Mass Index

Smoking was defined by whether the individual smoked at least 100 cigarettes in their lifetime, and was grouped as none, former, or current smoker. Physical activity was based on the individual’s engagement in work and recreational activities and was categorized as vigorous, moderate, and none. Obesity was determined using Body Mass Index(BMI) (kg/m^2^) and was calculated from physical examined weight and height. BMI was dichotomized as less than 25 or greater than or equal to 25.

#### Access to Health Care

Access to health care was defined by answers to the question “Is there a place that you usually go when you are sick or you need advice about your health?”. Those who answered ‘Yes’ or ‘there is more than one place’ were grouped as ‘Yes’; and those who answered ‘there is no place’ were grouped as ‘No’.

#### Demographic Variables

Demographic variables included gender, age (grouped as 20–34 years; 35–49 years; 50–64 years; and 65 + years), race/ethnicity (grouped as non-Hispanic White; non-Hispanic Black; Hispanic; and other Minority), education (dichotomized as high school or below and college or above), marital status (dichotomized as married or not married), and ratio of family income to poverty (dichotomized as 130% and less of poverty level and above 130% of poverty level).

#### Statistical Analysis

Data analysis was performed using SAS version 9.4 (SAS Institute). The data are weighted and the SURVEYFREQ, SURVEYMEANS, SURVEYREG, and SURVEYLOGISTIC procedures in SAS were used to represent the U.S. population and account for the complex survey design. The primary analytical goal was to explore the ACR and eGFR between US born and foreign born adults residing in the US. First, the nativity (US-born, foreign-born <15 years, foreign-born >=15 years) was compared by demographics using ANOVA. Second, US-born and foreign-born with >= 15 years of residence was compared by demographics using Rao-Scott chi-square statistics to determine whether the healthy immigrant effect hypothesis held. Third, the weighted means for ACR and eGFR were calculated for all the covariates. Fourth, ACR was dichotomized as greater than or equal to 30 mg/g and less than 30 mg/g, eGFR was dichotomized as less than 60 ml/min/1.73m^2^ and greater than or equal to 60 ml/min/1.73m^2^, univariate and multivariable survey logistic regression were used to assess the relationship between country of birth and ACR, country of birth and eGFR, controlling for length of time in the US. Last, demographic variables (including age, gender, race/ethnicity, education, marital status, and ratio of family income to poverty), access to health care, comorbidities, lifestyle factors were added in sequence to multivariable logistic regression to check for their influence. All p-values were 2-sided and p<.05 was considered statistically significant.

### Results

The population sample included 27,111 adults 20 years and older, representing 217,842,257 US adults 20 years and older. Table 1 provides weighted sample demographics. Approximately 26.1% reported being born outside of the US (foreign-born), with 40.4% of foreign-born having resided <15 years in the US. Among those reporting to be foreign-born with < 15 years of residence, 85% were under age 50, 51% were Hispanic, 44% earned below the poverty level based on 130% or less ratio of family income to the poverty line, 39.5% were not married, 54.4% had high school or below education. Comparatively, among individuals that were foreign-born and resided in the US ≥ 15 years, 52% were under age 50, 47% were Hispanic, 27.8% earned below the poverty level based on 130% or less ratio of family income to the poverty line, 37% were not married, 50% had high school or below education. Lastly, for US born, 19.6% were below the poverty level, 54.5% were married, 38.4% had high school or below education, 78.7% were NHW.

Table 2 provides information on the weighted percent of individuals with ACR <30 (low risk of poor outcomes) vs ≥ 30 (high risk of poor outcomes) and eGFR ≥ 60 (mild reduction of kidney function) vs <60 (severe reduction of kidney function) by country of birth and other covariates. Foreign-born had significantly lower percent of individuals with eGFR < 60 (3.61% for foreign born, 8.10% for US born, p<.0001). When considering length of time in the US, those with < 15 years in US had significantly lower percent of individuals with eGFR < 60 compared to those with 15 or more years in the US (1.14% for less than 15 years in US, 7.84% for 15 years and above in US, p<.0001). Individuals who had access to health care had significantly higher percent of individuals with ACR ≥ 30 (10.1% vs 6.6%; p<0.001) and a higher percent of individuals with eGFR <60 than those without access to health care (8.4% vs 1.3%; p<0.001), Individuals who had hypertension or MACE had significantly higher percent of individuals with ACR ≥ 30 and eGFR < 60 compared to those without hypertension or MACE. Information on the weighted means of ACR and eGFR by country of birth can be found in Appendix Table 1.

Table 3 presents the adjusted logistic regression analyses for the overall population. For the overall population, foreign-born were 26% more likely to have an ACR ≥ 30mg/g (OR=1.26, 95% CI: 1.08 – 1.47) adjusted by length of residence in US; 15% less likely to have an ACR ≥ 30mg/g (OR=0.85, 95%CI: 0.73 – 0.99) adjusted by length of residence and demographics. The model was no longer statistically significant after adding comorbidities. For the overall population, foreign-born were significantly less likely to have an eGFR < 60 when unadjusted (OR=0.42,95%CI: 0.36 – 0.50), and after adjusting for covariates. The odds ratio was 0.67 (95%CI: 0.56 – 0.79) when length of residence was added to the model; 0.76 (95% CI: 0.61 – 0.94) when length of residence, demographics, access of health care, and comorbidities were added to the model.

## Discussion

In a population representative of native-born and foreign-born adults in the United States, mean eGFR was high for both foreign-born (101.5) and US-born (92.9), while mean ACR was greater than 30 mg/mmol for both groups indicating possible presence of kidney damage [8]. As seen with other chronic diseases, foreign-born individuals who have resided in the US for 15 years or longer were more similar to the US-born population, with eGFR levels that were significantly lower than those who have resided in the US less than 15 years (108.9) and were about the same as the US-born population (92.9). While eGFR was greater than 60 for both groups, the decrease in eGFR seen in foreign-born who have resided in the US for 15 years or longer suggest a possible reduction in kidney function compared to newer immigrants.

While the unadjusted model comparing US-born to foreign-born for ACR > 30 was not statistically significant, after adjusting for length of residence the relationship became significant and showed that foreign-born individuals had 26% increased likelihood of having ACR > 30. This change in significance indicates that length of residence is an important factor to consider when explaining the relationship between nativity and risk of having an ACR >30. However, when the model was adjusted for length of residence, demographics, and access to care it was found that foreign-born had 15% less likelihood of having ACR >30 when. This shift in direction of association from increased risk to decreased risk indicates that demographic variables and access care are additional key drivers in explaining the relationship between ACR and nativity. Finally, once comorbidities were added to the model, the relationship was no longer significant which suggests that comorbidities may explain why length of residence in the US is an important factor in the relationship between immigration status and ACR.

The results showed that mean ACR was > 30 for both foreign-born and US-born individuals which poses an increased risk for all-cause and cardiovascular mortality [8]. Eventhough the mean eGFR was > 90 for both foreign-born and US born adults, these results indicate that both US-born and foreign-born individuals have some signs of kidney damage due to the elevated ACR seen in both groups of individuals. These results highlight the importance of having both eGFR and ACR values available before being able to declare absence of CKD.

This study provides previously unknown information about the relationship between foreign-born and US-born status and CKD as assessed by eGFR and ACR. Additional research needs to be done to gain further understanding on immigrant specific factors that may affect CKD development and progression in the foreign-born population residing in the US. It is known that immigrants often face multiple barriers to health care services including linguistic, transportation, financial, and cultural barriers [27, 28]. These barriers often negatively impact one’s ability to be screened for chronic disease, learn about risk factors, and to receive necessary treatment for disease management [29]. Our study also supports previous research that shows immigrant health status declines as length of US residence increases [13]. Based on the existing literature and our new findings, there is a need to increase screening for CKD in high risk US-born and foreign-born populations [30, 31], and long-term immigrants should be closely monitored for signs of kidney damage.

Strengths of this study include the large number of foreign-born and US-born individuals representative of adult population in the US, nine years of data, and availability of biological measures for CKD. The study does have a few noteworthy limitations. First, this was a cross-sectional study and causation cannot be determined based on the findings. In addition, we are unable to make a diagnosis of CKD in our study population since a decrease in eGFR (<60 ml/min/1.73m^2^) or evidence of kidney damage lasting three or more months is required. Second, there is possible recall bias due to self-report of variables other than ACR, eGFR, and BMI. Third, because the foreign-born population was slightly younger than the US-born our mean ACR and mean eGFR values may not reflect levels of older, high-risk individuals.

## Conclusions

This study fills an important gap in knowledge on the relationship between country of birth and signs of kidney damage. While foreign-born individuals had higher mean eGFR than US-born, foreign-born had no significant increase in odds of having an ACR greater than or equal to 30 mg/g after adjusting for all covariates. Foreign-born had lower odds of having eGFR less than 60 compared to US-born individuals, however, foreign-born with greater than 15 years in the US had mean values that were lower than those with less than 15 years in the US, and more similar to US-born individuals. Our results indicate there may be some decrease in kidney function as the length of US residence increases, however, more research is needed to gain a greater understanding of the relationship. Based on these results efforts to increase access to screening and awareness of CKD may be warranted for immigrant populations.

## Supplementary Material

10903_2020_1054_MOESM1_ESM

## Figures and Tables

**Table 1:** Weighted sample demographics of adults ≥20 in US-born and foreign-born (FB)

	US-Born Sample n=20,048	FB< 15 years in the US Sample n=2,852	FB>= 15 years in the US Sample n= 4,211	US-Born vs FB <15 vs FB >= 15 years in the US p-value	US Born vs FB >= 15 years in the US p-value
**Gender**				0.0020	0.1218
Male	47.67%	51.13%	49.01%		
Female	52.33%	48.87%	50.99%		
**Age group**				<.0001	<.0001
20–34 yrs	27.21%	49.01%	15.18%		
35–49 yrs	27.48%	35.96%	36.83%		
50–64 yrs	26.49%	10.42%	30.19%		
65 + yrs	18.82%	4.61%	17.80%		
**Race**				<.0001	<.0001
Non-Hispanic White	78.65%	15.41%	20.83%		
Non-Hispanic Black	12.34%	7.55%	6.78%		
Hispanic	5.92%	50.56%	47.08%		
other Minority	3.09%	26.47%	25.31%		
**Education level**				<.0001	<.0001
High school or below	38.35%	54.42%	50.33%		
College or above	61.65%	45.58%	49.67%		
**Marital status**				<.0001	<.0001
Married	54.54%	60.53%	63.01%		
not Married	45.46%	39.47%	36.99%		
**Ratio of family income to poverty**				<.0001	<.0001
130% and less of poverty level	19.56%	44.24%	27.77%		
above 130% of poverty level	80.44%	55.76%	72.23%		
**Access to health care**				<.0001	<.0001
No	12.29%	35.98%	17.95%		
Yes	87.71%	64.02%	82.05%		
**Hypertension**				<.0001	0.0012
No	66.57%	86.78%	70.88%		
Yes	33.43%	13.22%	29.12%		
**MACE**				<.0001	<.0001
No	90.71%	97.24%	93.10%		
Yes	9.29%	2.76%	6.90%		
**Depression**				<.0001	<.0001
None depression	84.74%	77.41%	81.10%		
Depression	7.22%	4.92%	6.22%		
Unknown	8.04%	17.67%	12.68%		
**BMI categories**				<.0001	0.2799
BMI < 25	30.40%	39.83%	31.91%		
BMI >= 25	69.60%	60.17%	68.09%		
**Smoking status**				<.0001	<.0001
None Smoker	51.67%	71.17%	65.87%		
Current Smoker	22.92%	13.94%	12.85%		
Former Smoker	25.40%	14.89%	21.29%		
**Physical activity**				<.0001	<.0001
None	28.83%	36.48%	36.49%		
Vigorous	38.36%	37.23%	31.74%		
Moderate	32.81%	26.29%	31.77%		

**Table 2:** Weighted Sample Percents of Individuals with Albumin-to-Creatinine Ratio and Estimated Glomerular Filtration Rate

	Albumin-to-Creatinine Ratio			Estimated Glomerular Filtration Rate		
	ACR <30	ACR >=30	P Value	eGFR >=60	eGFR <60	P Value
Overall	90.41%	9.59%		92.67%	7.33%	
**Birth of country**			0.3856			<.0001
U.S.-born	90.50%	9.50%		91.90%	8.10%	
Foreign-born	90.00%	10.00%		96.39%	3.61%	
**Length of time in US**			0.0285			<.0001
<15 years	91.74%	8.26%		98.86%	1.14%	
>=15 years	90.27%	9.73%		92.16%	7.84%	
**Gender**			0.0002			<.0001
Male	91.27%	8.73%		93.78%	6.22%	
Female	89.61%	10.39%		91.63%	8.37%	
**Age group**			<.0001			<.0001
20–34 yrs	94.69%	5.31%		99.85%	0.15%	
35–49 yrs	92.40%	7.60%		98.89%	1.11%	
50–64 yrs	90.92%	9.08%		94.06%	5.94%	
65 + yrs	79.42%	20.58%		69.08%	30.92%	
**Race**			<.0001			<.0001
Non-Hispanic White	91.23%	8.77%		91.35%	8.65%	
Non-Hispanic Black	87.23%	12.77%		93.32%	6.68%	
Hispanic	89.14%	10.86%		96.97%	3.03%	
other Minority	90.02%	9.98%		96.43%	3.57%	
**Education level**			<.0001			<.0001
High school or below	87.71%	12.29%		90.81%	9.19%	
College or above	92.25%	7.75%		93.94%	6.06%	
**Marital status**			<.0001			0.1983
Married	91.27%	8.73%		92.90%	7.10%	
not Married	89.30%	10.70%		92.36%	7.64%	
**Ratio of family income to poverty**			<.0001			0.6741
130% and less of poverty level	86.65%	13.35%		92.60%	7.40%	
above 130% of poverty level	91.63%	8.37%		92.81%	7.19%	
**Access to health care**			<.0001			<.0001
No	93.39%	6.61%		98.73%	1.27%	
Yes	89.90%	10.10%		91.63%	8.37%	
**Hypertension**			<.0001			<.0001
No	93.67%	6.33%		96.86%	3.14%	
Yes	83.30%	16.70%		83.57%	16.43%	
**MACE**			<.0001			<.0001
No	91.80%	8.20%		94.73%	5.27%	
Yes	75.00%	25.00%		70.46%	29.54%	
**Depression**			<.0001			0.8619
None depression	90.89%	9.11%		92.63%	7.37%	
Depression	87.05%	12.95%		92.87%	7.13%	
Unknown	88.32%	11.68%		92.89%	7.11%	
**BMI categories**			0.0126			<.0001
BMI < 25	91.33%	8.67%		94.29%	5.71%	
BMI >= 25	90.24%	9.76%		92.12%	7.88%	
**Smoking status**			<.0001			<.0001
None Smoker	91.36%	8.64%		93.02%	6.98%	
Current Smoker	90.30%	9.70%		96.63%	3.37%	
Former Smoker	88.40%	11.60%		88.45%	11.55%	
**Physical activity**			<.0001			<.0001
None	85.49%	14.51%		87.39%	12.61%	
Vigorous	93.89%	6.11%		97.07%	2.93%	
Moderate	90.87%	9.13%		92.36%	7.64%	

**Table 3.** Survey Multivariable Logistic Analysis for Albumin-to-Creatinine Ratio and Estimated Glomerular Filtration Rate Adjusted for Length of Residence, Demographic Variables, Access to Health Care, Comorbidities, and Lifestyle Factors

		Albumin-to-Creatinine Ratio		Estimated Glomerular Filtration Rate	
	Place of Birth	Odds Ratio (95% CI)	P Value	Odds Ratio (95% CI)	P Value
Model 1	Foreign-born	1.06(0.93 – 1.20)	0.3897	**0.42(0.36** – **0.50)**	**<.0001**
Model 2	Foreign-born	**1.26(1.08** – **1.47)**	**0.0031**	**0.67(0.56** – **0.79)**	**<.0001**
Model 3	Foreign-born	**0.85(0.73** – **0.99)**	**0.0361**	**0.68(0.55** – **0.85)**	**0.0006**
Model 4	Foreign-born	**0.85(0.73** – **1.00)**	**0.0442**	**0.69(0.56**–**0.85)**	**0.0006**
Model 5	Foreign-born	0.93(0.80 – 1.09)	0.3713	**0.76(0.61** – **0.94)**	**0.0120**
Model 6	Foreign-born	0.95(0.81 – 1.11)	0.5085	**0.75(0.61** – **0.93)**	**0.0090**

Reference groups: Place of Birth (US born); ACR (<30); eGFR (>=60)

Model 1: unadjusted model comparing Foreign born vs US born

Model 2: model 1 + length of residence

Model 3: model 2 + demographic variables (gender, age, race/ethnicity, education, marital status, ratio of family income to poverty)

Model 4: model 3 + access to health care

Model 5: model 4 + comorbidities (hypertension, major adverse cardiac event (MACE), depression

Model 6: model 5 + lifestyle factors (smoking, physical activity, body mass index (BMI)
