# Supplementary material for: The relationship between immigration status and chronic kidney disease risk factors in immigrants and US-born adults
Source: J Immigr Minor Health. Author manuscript; Available in PMC 2021 Dec 1. (PMC7686246; doi:10.1007/s10903-020-01054-x)
Supplement: 10903_2020_1054_MOESM1_ESM [file NIHMS1613207-supplement-10903_2020_1054_MOESM1_ESM.docx]

Appendix Table 1: Weighted Sample Means for ACR and eGFR

|  | **Albumin Creatinine Ratio** | | **Estimated Glomerular Filtration Rate** | |
| --- | --- | --- | --- | --- |
|  | Mean (95% CI) | P Value | Mean (95% CI) | P Value |
| **Country of birth** |  | 0.0452 |  | <.0001 |
| U.S.-born | 22.06 (20.69 - 23.43) |  | 91.92 (91.17 - 92.67) |  |
| Foreign-born | 24.73 (22.48 - 26.99) |  | 101.49 (100.61 - 102.38) |  |
| **Length of time in US** |  | 0.3934 |  | <.0001 |
| <15 years | 21.25 (18.36 - 24.13) |  | 108.87 (107.89 - 109.86) |  |
| >=15 years | 22.66 (21.33 - 23.98) |  | 92.29 (91.59 - 92.99) |  |
| **Gender** |  | 0.3734 |  | 0.0547 |
| Male | 21.97 (20.34 - 23.60) |  | 93.24 (92.55 - 93.93) |  |
| Female | 23.03 (21.28 - 24.79) |  | 93.85 (93.02 - 94.68) |  |
| **Age group** |  | <.0001 |  | <.0001 |
| 20-34 yrs | 12.78 (11.59 - 13.97) |  | 112.15 (111.43 - 112.87) |  |
| 35-49 yrs | 18.72 (16.71 - 20.73) |  | 98.39 (97.78 - 98.99) |  |
| 50-64 yrs | 24.23 (21.59 - 26.88) |  | 85.65 (84.98 - 86.31) |  |
| 65 + yrs | 42.02 (39.11 - 44.92) |  | 68.22 (67.59 - 68.85) |  |
| **Race** |  | <.0001 |  | <.0001 |
| Non-Hispanic White | 19.74 (18.34 - 21.13) |  | 89.64 (88.90 - 90.38) |  |
| Non-Hispanic Black | 32.22 (28.65 - 35.78) |  | 101.14 (100.01 - 102.27) |  |
| Hispanic | 26.44 (23.91 - 28.97) |  | 104.67 (103.79 - 105.56) |  |
| other Minority | 26.44 (21.41 - 31.47) |  | 99.21 (97.96 - 100.45) |  |
| **Education level** |  | <.0001 |  | 0.6150 |
| High school or below | 28.20 (26.02 - 30.37) |  | 93.70 (92.84 - 94.56) |  |
| College or above | 18.65 (17.33 - 19.97) |  | 93.47 (92.65 - 94.28) |  |
| **Marital status** |  | 0.0080 |  | <.0001 |
| Married | 21.10 (19.51 - 22.68) |  | 91.26 (90.51 - 92.00) |  |
| not Married | 24.34 (22.54 - 26.15) |  | 96.53 (95.72 - 97.35) |  |
| **Ratio of family income to poverty** |  | <.0001 |  | <.0001 |
| 130% and less of poverty level | 30.48 (27.17 - 33.79) |  | 98.72 (97.51 - 99.93) |  |
| above 130% of poverty level | 19.97 (18.73 - 21.21) |  | 92.09 (91.42 - 92.77) |  |
| **Access to health care** |  | <.0001 |  | <.0001 |
| No | 15.33 (13.64 - 17.02) |  | 104.05 (103.13 - 104.96) |  |
| Yes | 23.76 (22.33 - 25.18) |  | 91.76 (91.06 - 92.46) |  |
| **Hypertension** |  | <.0001 |  | <.0001 |
| No | 14.41 (13.56 - 15.26) |  | 98.59 (97.90 - 99.27) |  |
| Yes | 40.27 (37.11 - 43.44) |  | 82.61 (81.76 - 83.46) |  |
| **MACE** |  | <.0001 |  | <.0001 |
| No | 19.09 (18.08 - 20.11) |  | 95.48 (94.79 - 96.17) |  |
| Yes | 60.46 (52.36 - 68.56) |  | 72.86 (71.68 - 74.04) |  |
| **Depression** |  | <.0001 |  | <.0001 |
| None depression | 21.05 (19.86 - 22.24) |  | 93.25 (92.51 - 93.99) |  |
| Depression | 34.54 (29.41 - 39.66) |  | 94.04 (92.81 - 95.28) |  |
| Unknown | 27.30 (23.72 - 30.89) |  | 96.44 (91.18 - 97.71) |  |
| **BMI** |  | 0.0293 |  | <.0001 |
| BMI < 25 | 20.43 (19.04 - 21.82) |  | 96.60 (95.69 - 97.50) |  |
| BMI >= 25 | 22.61 (21.07 - 24.15) |  | 92.31 (91.61 - 93.00) |  |
| **Smoking status** |  | <.0001 |  | <.0001 |
| None smoker | 20.23 (18.67 - 21.79) |  | 94.53 (93.66 - 95.41) |  |
| Current smoker | 23.29 (21.35 - 25.23) |  | 99.04 (98.29 - 99.79) |  |
| Former smoker | 26.94 (24.25 - 29.63) |  | 86.64 (85.70 - 87.59) |  |
| **Physical activity** |  | <.0001 |  | <.0001 |
| None | 33.71 (31.16 - 36.26) |  | 89.96 (89.07 - 90.84) |  |
| Vigorous | 14.78 (13.46 - 16.09) |  | 97.62 (96.73 - 98.52) |  |
| Moderate | 21.25 (19.39 - 23.12) |  | 92.10 (91.27 - 92.94) |  |
